# Elucidation of molecular function of phage protein responsible for optimization of host cell lysis

**DOI:** 10.1186/s12866-024-03684-9

**Published:** 2024-12-19

**Authors:** Jinwoo Kim, Joonbeom Kim, Sangryeol Ryu

**Affiliations:** 1 Department of Food and Animal Biotechnology, Research Institute of Agriculture and Life Sciences, Seoul National University, Seoul, Republic of Korea; 2 Department of Agricultural Biotechnology, College of Agriculture and Life Sciences, Seoul National University, Seoul, Republic of Korea; 3 Center for Food and Bioconvergence, Seoul National University, Seoul, Republic of Korea

**Keywords:** Bacteriophages, Superinfection, Holin, Anti-holin, Lysis inhibition

## Abstract

**Background:**

Bacteriophages (or phages) replicate by utilizing bacterial resources and destroy their host cells at the end of the replication cycle. Phages employ multiple proteins to optimize host cell lysis, thereby maximizing the production of phage particles. However, elucidating the entire lysis process is challenging due to the abundance of uncharacterized genes in the phage genome.

**Results:**

In this study, we identified a gene *orf52* from BSPM4 phage genome that showed antibacterial activity in *Salmonella*. Investigation of physiological role of ORF52 in the phage replication revealed that ORF52 could modulate the holin function to fine-tune a cell lysis, providing replication advantages to phages under high phage population density.

**Conclusions:**

We concluded that ORF52 may optimize phage replication by modulating the timing of phage-mediated cell lysis. This study provides a unique example of a phage protein involved in fine-tuning lysis timing.

**Supplementary Information:**

The online version contains supplementary material available at 10.1186/s12866-024-03684-9.

## Background

Bacteriophages (or phages) are viruses that specifically infect host bacteria and constitute the most ubiquitous entities on Earth. As obligate intracellular parasites, phages rely on host machinery to replicate and produce viral progeny. Phage infections begin with the attachment (adsorption) to specific receptors on the host bacteria, followed by the injection of their genetic material into the bacterial cytoplasm [1]. They hijack the host’s molecular machinery for their own replication and eventually lyse the host bacteria to release newly synthesized viral particles. Due to their host specificity and lytic activity, phages have a significant impact on shaping the structure of bacterial communities by altering species abundance [2]. Furthermore, since phages can mediate horizontal gene transfer and modify the bacterial genome through the integration of a phage genome into the bacterial chromosome, they play a crucial role in driving bacterial evolution [3]. Phages can be found in diverse environments, including air, water, soil, animal, plant, and even ancient fossilized stool specimen [4, 5]. The estimated number of phages on Earth is 10^31^, which might be ten times higher than that of bacteria [6–9]. The ecological significance of phages can be attributed to their abundance and diversity in the environment [10].

Advances in whole-genome sequencing technologies have led to the identification of numerous phage genomes, providing deep insight into the genetic diversity of phages [11]. To date (March 2024), approximately 14,000 completely sequenced phage genomes are available in the GenBank database. Recent phage genomic studies have reinforced the view that phages are extremely diverse and constitute enormous sources of uncharacterized genes [12, 13]. Phage genome sizes range from 2,435 bp (*Leuconostoc* phage L5) to 540 kbp (*Prevotella* mega-phage Lak), reflecting the tremendous diversity of the phage population [14]. Furthermore, approximately 70% of phage-derived proteins do not have bioinformatically predicted functions [15, 16]. Given the vast number of phages and their significant effects on the environment, elucidation of phage gene function is important for an understanding of microbial biology and for the efficient exploitation of phages as biocontrol agents for human benefit.

Phage BSPM4 is a double-stranded DNA virus that specifically infects *Salmonella*. The majority of genes encoded in the phage BSPM4 genome are annotated as hypothetical proteins, accounting for 75.6% of the total genes. BSPM4 possesses a structurally distinct endolysin with a transmembrane domain at the C-terminus [17, 18]. A previous study reported that the BSPM4 endolysin could induce cell lysis without the help of holin when it was overexpressed in vitro [18]. These unique features led us to explore the genome of phage BSPM4 that might employ an unusual lysis mechanism that differs from the other phages. Screening of a random genomic library of BSPM4 revealed that expression of the *orf52* gene was lethal to *Salmonella* even though the ORF52 does not share amino acid sequence homology with any characterized protein. We found that the ORF52 plays an important role in phage-mediated cell lysis by modulating the function of holin, suggesting that ORF52 might be an anti-holin that can modulate the timing of phage-mediated cell lysis to maximize the production of viral progeny.

## Methods

### Bacterial strains, plasmids, and growth conditions

The bacterial strains were grown in Luria-Bertani (LB) medium or agar. When necessary, the media and agars are supplemented with antibiotics or chemical agents at the following concentrations, kanamycin (Km, 50 µg/mL), chloramphenicol (Cm, 12.5 µg/mL), ampicillin (Ap, 50 µg/mL), isopropyl-*β*-thiogalactopyranoside (IPTG), and L-(+)-arabinose. Plasmids used in this study are listed in Table S1.

### Construction of phage DNA library

The genomic DNA from phage BSPM4 was isolated by phenol-chloroform extraction method. Phage lysates were treated with RNase (1 µg/mL) and DNase (1 µg/mL) for 30 min at 25 °C to remove non-phage DNA or RNA. Following the addition of proteinase K (50 µg/mL), ethylene-diamine-tetraacetic acid (EDTA, 20 mM; pH 8.0), and sodium dodecyl sulfate (SDS, 0.5% [wt/vol]), the lysates were incubated at 56 °C for 1 h. The extracted DNA was purified using phenol-chloroform and precipitated with ethanol. The phage DNA was randomly digested with restriction enzyme EcoRI and ligated into the equally digested vector pUHE21-2-lacI^q^. The vector libraries were transformed into *E. coli* DH5α.

### Screening of antibacterial phage proteins

The resultant clones (267 colonies) from random library were picked and grown overnight in 96-well plate in LB medium. The cultures were diluted 1:50 in fresh LB broth containing 0.5mM IPTG and grown at 37℃ for 12 h. We measured the absorbance of the cultures at 600 nm at the endpoint and selected clones with significantly reduced bacterial growth when compared to a negative control containing a backbone plasmid. To further verify the antibacterial effects of inserted phage DNA, the growth of the primary selected clones (24 colonies) was monitored by measuring absorbance at 600 nm and one clone showing rapid cell lysis by expression of cloned phage gene was selected.

### Bacterial membrane fraction

Bacterial cells harboring p*orf52-his*_*x6*_ were grown in LB medium until the optical density at 600 nm reached approximately 1.0 and then treated with 0.5 mM of IPTG. Bacterial cells were harvested and resuspended with buffer (20 mM tris; pH 6.8). The suspensions were disrupted by sonication and used as whole cell lysate (W). Subsequently, cell debris were removed by centrifugation at 12,000 x g for 30 min. The resultant supernatant was ultra-centrifuged at 100,000 x g for 60 min. The resulting supernatant was used as a cytoplasmic fraction (C), and the pellet was used as a membrane fraction (M).

### Western blot

The prepared samples were separated using sodium dodecyl sulfate-polyacrylamide gel electrophoresis (SDS-PAGE). They were transferred to a polyvinylidene difluoride membrane. The transferred membrane was blocked with 5% of non-fat dry milk and probed with anti-DnaK (1: 10,000 dilutions; Enzo life sciences, USA), anti-OmpA (1: 10,000 dilutions; Antibody research corporation, USA), anti-His antibody (1: 3,000 dilutions; Sigma-Aldrich, USA), and anti-FLAG antibody (1: 5,000 dilutions; Sigma-Aldrich, USA) as primary antibodies. A secondary antibody, anti-mouse IgG conjugated to peroxidase (diluted at 1:5,000; GenDEPOT, USA), was employed in the experiment. Chemiluminescent signals were developed using ECL reagent (Promega, USA) and detected by ChemiDoc (Bio-Rad, USA).

### SYTOX green assay

Membrane disruption caused by the expression of *orf52* was monitored using a Sytox green nucleic acid stain dye (Thermofisher, USA). Bacterial cells harboring p*orf52* were exponentially grown in LB medium and treated with 1 µM SYTOX green dye. After incubation of the culture for 30 min at 37℃ in the dark, the suspensions were dispensed into wells of 96-well plate and treated with 0.1mM IPTG for inducing *orf52* gene. Fluorescence was quantified using a spectrophotometer (Molecular Devices, USA) by detecting signals at an excitation wavelength of 485 nm and an emission wavelength of 522 nm.

### Co-immunoprecipitation

*Salmonella* cells harboring pUHE21-2-lacI^q^::*flag-orf37*(FLAG-ORF37*)* and pBAD33::*orf52-his*_*x6*_ (ORF52-his_x6_) were grown in LB and the protein(s) were expressed by adding 0.5 mM of IPTG or/and 0.2% of arabinose. The bacterial cells suspended in buffer (20 mM tris and 30% glycerol; pH 6.8) were disrupted by sonication. After centrifugation (15,000 x g, for 30 min, at 4℃), the supernatant was passed through a Ni-nitrilotriacetic acid (Ni-NTA) superflow column (Qiagen, Germany). The purification of his-tagged proteins was conducted according to the manufacturer’s instruction. The column was washed four times with 10 ml of washing buffer (20 mM tris and 20 mM imidazole; pH 6.8). The proteins were eluted and analyzed by western blot assay.

### Phage engineering

For constructing mutant phages lacking *orf52* gene, we used a type II CRISPR-Cas9 system. The immediate upstream and downstream regions of *orf52* gene, respectively, were amplified by PCR. The resulting PCR products were cloned into a pT plasmid and served as templates for homologous recombination. The template vector (pT-*orf52*) and CRISPR-Cas9 vector targeting the *orf52* gene (pCas9-*orf52*) were co-transformed into *Salmonella* LT2(c). The bacterial cells containing template vector (pT-*orf52*) and CRISPR/Cas9 vector were grown at 30℃ until the OD_600_ reached approximately 1.5 and infected with phage BSPM4 at MOI of 10^− 6^. The culture was incubated at 30℃ for 12 h. The phage lysate was plated on *Salmonella* containing pCas9-*orf52*. The plaques were picked and suspended in sterilized water. Deletion of target gene was confirmed by PCR and sequencing.

### One-step growth curve

The *Salmonella* LT2(c) culture was cultured until it reached the mid-exponential phase. Phages were added at a MOI of 0.001 for 5 min at 37℃. The phage-host mixture was centrifuged at 15,000 x g for 5 min. The resulting pellet was resuspended in fresh LB medium. The suspension was incubated at 37℃. Samples were taken every 10 min. The number of plaque forming unit (PFU) in taken samples was immediately determined.

### Quantification and statistical analysis

All statistical analysis details, such as the number of repeats (n), p-values, and types of statistical tests employed, are provided within the figure legends. If not specified, *n* = 3. Graphs represent the mean value +/- the SD. Statistical comparison between two groups was conducted using either the Student’s t-test or the Welch’s t-test, depending on the homogeneity of variances as determined by Levene’s test. When comparing more than two groups, statistical significance was evaluated using one-way analysis of variance, followed by the Bonferroni post-hoc test for multiple comparisons.

## Results and discussion

### Distinct bacterial cell lysis system of phage BSPM4

BSPM4 is a double-stranded DNA phage that specifically targets *Salmonella* [17]. Typically, double-stranded DNA phages use a holin-endolysin system to accomplish host cell lysis [19, 20]. In the canonical holin-endolysin system, the holins induce the formation of pores and allow the passage of the cytosol-accumulated endolysins to the peptidoglycan layer [21]. In non-canonical systems, endolysins have a signal-arrest-release (SAR) domain [22]. The SAR endolysins remain tethered to the cytoplasmic membrane in an inactive form until holins (or pinholins) dissipate the proton motive force [23]. Reported SAR endolysins possess a transmembrane domain at the N-terminus and are secreted to the periplasm via the general secretory (Sec) pathway [22]. However, the BSPM4 endolysin (ORF38) harbors a putative transmembrane domain at the C-terminus and lacks a predicted signal peptide sequence (Fig. S1). Moreover, the Sec pathway does not play a role in endolysin-mediated cell lysis in phage BSPM4 [18]. These unusual features led us to investigate the host cell lysis system of phage BSPM4.

### ORF52 from the phage BSPM4 can induce bacterial cell lysis

**Fig. 1:**
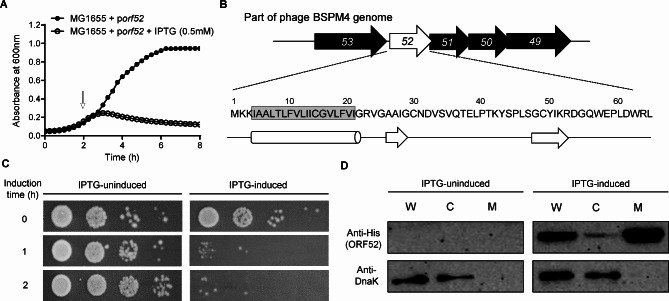
Phage protein ORF52 involved in bacterial cell lysis. (**A**) Growth of *E. coli* MG1655 harboring the phage gene *orf52* (p*orf52*) was monitored by measuring its absorbance at 600 nm. The arrow indicates the time point of 0.5mM IPTG addition. (**B**) Primary and secondary structure of phage protein ORF52 were predicted. The potential membrane-spanning helix is highlighted in grey. Arrows mark the β-strands, and the cylinder indicates α-helix. (**C**) *E. coli* transformed with a plasmid encoding the ORF52 (p*orf52*) were grown with or without 0.5 mM IPTG, and the CFUs were determined at the indicated time points. (**D**) *E. coli* harboring the plasmid encoding a C-terminal His-tagged ORF52 (pUHE21::*orf52-his*_*x6*_) was cultured and fractionated into whole cell lysate [W], cytoplasm [C], and membrane [M] fractions. DnaK was used as the control for cytoplasmic fractions. The uncropped western blot data are shown in Fig. S9

The BSPM4 genome is 59,097 bp with a G + C content of 56.5% [17]. It contains a total of 78 putative open reading frames (ORFs), of which 19 ORFs (24.4%) are predicted to have functional domains. The remaining 59 ORFs (75.6%) have been annotated as hypothetical proteins. To identify antibacterial gene(s) from the BSPM4 genome, we constructed a random DNA library and screened 267 clones for antibacterial activity. One clone showed strong growth inhibition upon induction in *Salmonella* and analysis of the inserted phage DNA sequences in the clone revealed the presence of five ORFs; *orf53*, *orf52*, *orf51*, *orf50*, and *orf49* (Fig. S2A). As the expression of the *orf52* gene induced the strongest bacterial cell lysis among these five genes, we focused on *orf52* for further study (Fig. 1A and C, and Fig. S2B). The phage *orf52* gene encodes a 64-amino-acid protein with a transmembrane domain at the N-terminus [24] (Fig. 1B). Bioinformatic analysis revealed that many genes homologous to the *orf52* are present in the phages with a genome organization similar to BSPM4, including phage Chi, iEPS5, FSLSP030, and BP12C (Fig. S2C). However, the protein ORF52 does not have apparent sequence homology to any protein with known function.

### Membrane disruption caused by ORF52

**Fig. 2:**
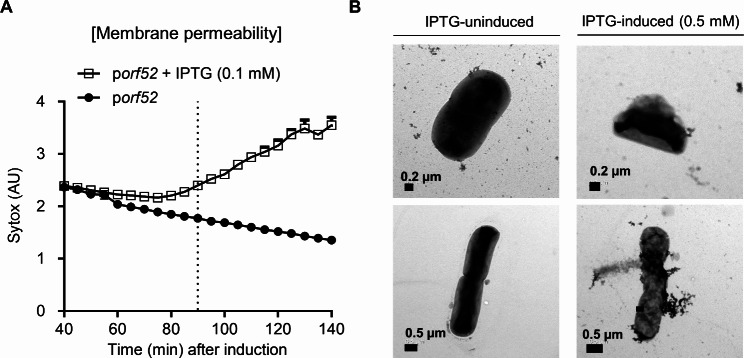
Membrane damages caused by expression of ORF52. (**A**) Membrane permeability of cells expressing *orf52* was assessed using the Sytox green dye. The dashed line indicates the point at which cell viability starts to decrease. Fluorescence intensity is expressed in arbitrary units (AU). (**B**) Morphological changes to bacterial cells transformed with a plasmid encoding the ORF52 (p*orf52*) were observed at 1 h after 0.5 mM IPTG induction. Scale bars are indicated as black lines in the lower left corner

Since the ORF52 is predicted to possess a single transmembrane domain at the N-terminus, we investigated its cellular localization in bacteria. Analysis of cytoplasmic and membrane fractions of the bacterial cells expressing the His-tagged ORF52 showed that significant amounts of ORF52 were found in the membrane fraction (Fig. 1D). As ORF52 can be localized to the cell membrane, we hypothesized that the expression of the *orf52* gene might affect the integrity of the bacterial cell membrane, ultimately leading to cell death. We assessed bacterial membrane permeability by measuring the uptake of the Sytox green dye. Surge in the fluorescent signal was observed preceding the decrease of cell viability in the bacterial cells expressing *orf52* (Fig. 2A), indicating increased membrane permeability due to membrane damage by the ORF52. Transmission electron microscopy (TEM) analysis of bacterial membrane also revealed that bacterial cells expressing the *orf52* showed apparent membrane damage compared to the bacterial cells with no *orf52* expression (Fig. 2B). These findings suggest that changes in membrane permeability caused by the overexpression of ORF52 could disrupt the integrity of the bacterial membrane, ultimately resulting in cell death.

### Effects of ORF52 on function of phage lysis cassette

**Fig. 3:**
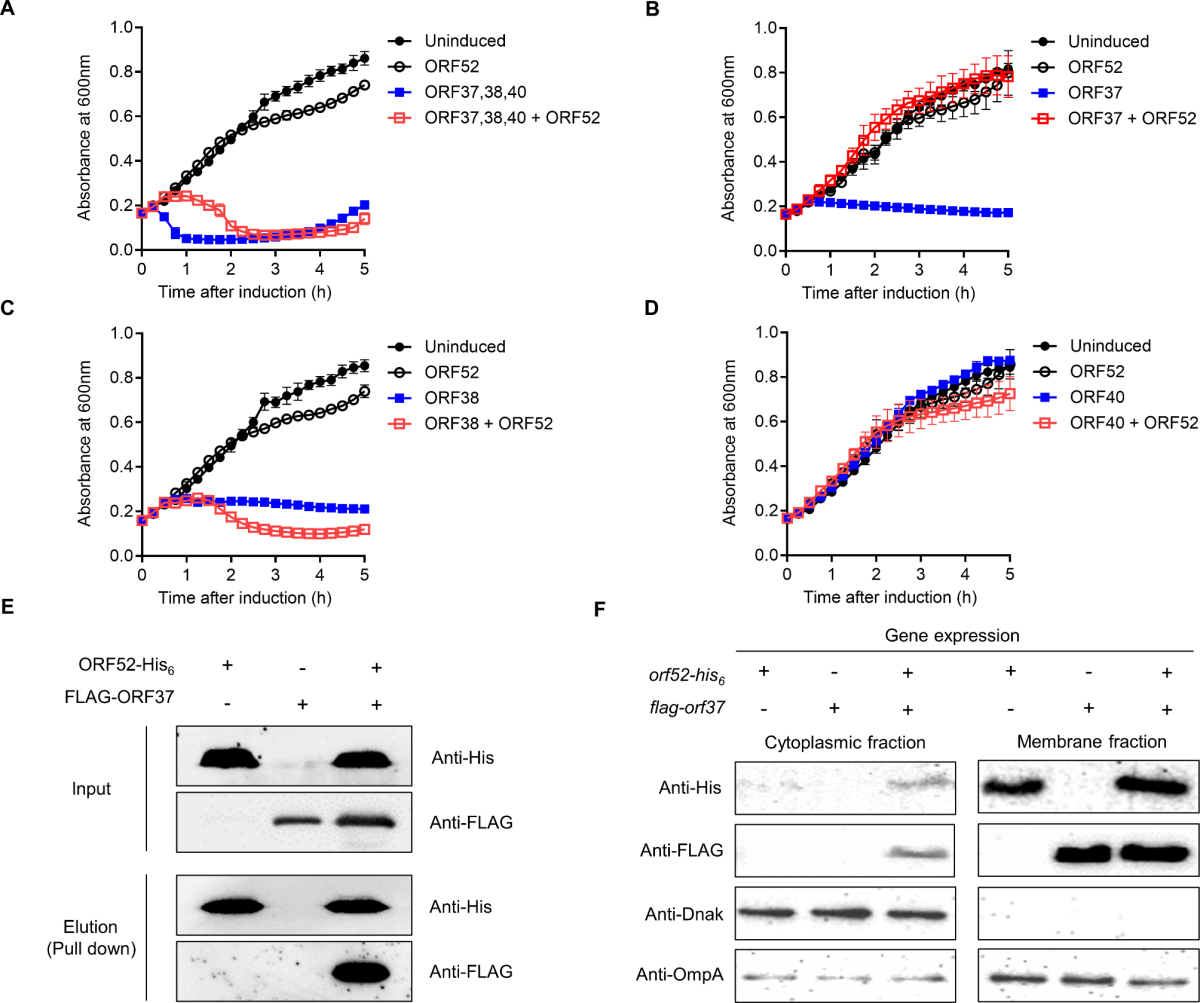
Effects of ORF52 on phage lysis. The bacteria harbored two plasmids encoding the *orf52* and phage lysis gene(s), including *orf37*,*38*,*40* (**A**), *orf37* (**B**), *orf38* (**C**), and *orf40* (**D**), The expression of *orf52* (pBAD-o*rf52*) was induced by 0.02% arabinose and that of phage lysis gene(s) (porf*) was induced by 0.5mM IPTG. Bacterial cells expressing *orf52* or orf* were designated as ORF52 or ORF*. The cell expressing both *orf52* and orf* was designated as ORF52 + ORF*. (**E**) The interaction between ORF52 and ORF37 was determined by co-immunoprecipitation experiment. Full-length blots are presented in Fig. S10. (**F**) The *Salmonella* Typhimurium harboring the two plasmids encoding an N-terminal FLAG-tagged ORF37 (pUHE1::*flag-orf37*) and C-terminal His-tagged ORF52 (pBAD33::*orf52-hisx6*) were cultured. After expressing ORF52 for 20 min, ORF37 was subsequently induced for 20 min. The cultures were then fractionated into cytoplasmic and membrane fractions. DnaK was used as the control for the cytoplasmic fractions, while OmpA served as the control for the membrane fractions. Fig. S11 presents the western blot data in its uncropped form

The phage BSPM4 genome encodes three classical lysis cassette genes, including *orf37* (putative holin), *orf38* (putative endolysin), and *orf40* (putative spanin) (Fig. S1). To determine whether ORF52 could affect the function of the lysis cassette protein(s), we co-expressed *orf52* and the lysis cassette gene(s) in *Salmonella* and monitored bacterial growth by measuring absorbance at 600 nm. Since overexpression of *orf52* alone can cause rapid cell lysis, we induced the expression of *orf52* at a level that did not affect bacterial cell growth using a low-copy number plasmid (pBAD33::*orf52*) with a low concentration of inducer (0.02% arabinose). When whole lysis cassette, including holin (ORF37), endolysin (ORF38), and spanin (ORF40), was simultaneously expressed, bacterial cells lysed rapidly within 15 to 30 min. However, co-expression of the entire lysis cassette genes and the *orf52* resulted in delayed bacterial lysis (Fig. 3A). To identify the gene(s) affected by ORF52, we subcloned the holin, endolysin, and spanin separately and analyzed the effects of *orf52* expression on each of these genes (Fig. 3B-D). When the BSPM4 endolysin was simultaneously expressed with ORF52, a synergistic antibacterial effect was observed (Fig. 3C). This could be attributed to ORF52’s potential to increase the permeability of bacterial membranes, thereby facilitating the access of endolysins to the peptidoglycan layer. However, ORF52 completely suppressed the bacterial growth inhibition induced by holin (Fig. 3B and Fig. S3).

### Interaction between ORF52 and ORF37

As ORF52 expression caused a delay in bacterial cell lysis triggered by the classical phage lysis cassettes (Fig. 3A), we explored the potential role of ORF52 in inhibiting the antibacterial activity of ORF37 (holin). Given that both ORF37 and ORF52 can be localized to the bacterial membrane, there is a possibility that ORF52 might inhibit holin function of ORF37 by forming a protein complex. Indeed, the protein complex formation between ORF52 and ORF37 was seen with a pull-down assay using lysates from cells expressing both ORF52 fused with a His-tag at the C-terminus (ORF52-his_x6_) and ORF37 fused with a FLAG-tag at the N-terminus (FLAG-ORF37) (Fig. 3E). These results imply that ORF52 can inhibit the antibacterial function of ORF37 by either disturbing the membrane localization of ORF37 or inhibiting oligomerization of ORF37 within the bacterial membrane. We investigated the effect of ORF52 expression on the cellular localization of ORF37 (Fig. 3F and Fig. S4). Most of ORF52 and ORF37 were localized on the membrane fraction when each of this protein was expressed separately as shown on Fig. 3F. Nearly similar pattern was observed when ORF52 was co-expressed with ORF37 even though very small amount of ORF37 and ORF52 was detected in the cytoplasmic fraction. Cell lysis activities of ORF37 and ORF52 was reduced when both ORF37 and ORF52 were co-expressed (Fig. 3B), suggesting that the primary mechanism for anti-holin action of ORF52 might be associated with its interaction with ORF37 within the membrane.

### Characterization of wild-type and mutant phage lacking *orf52*

**Fig. 4:**
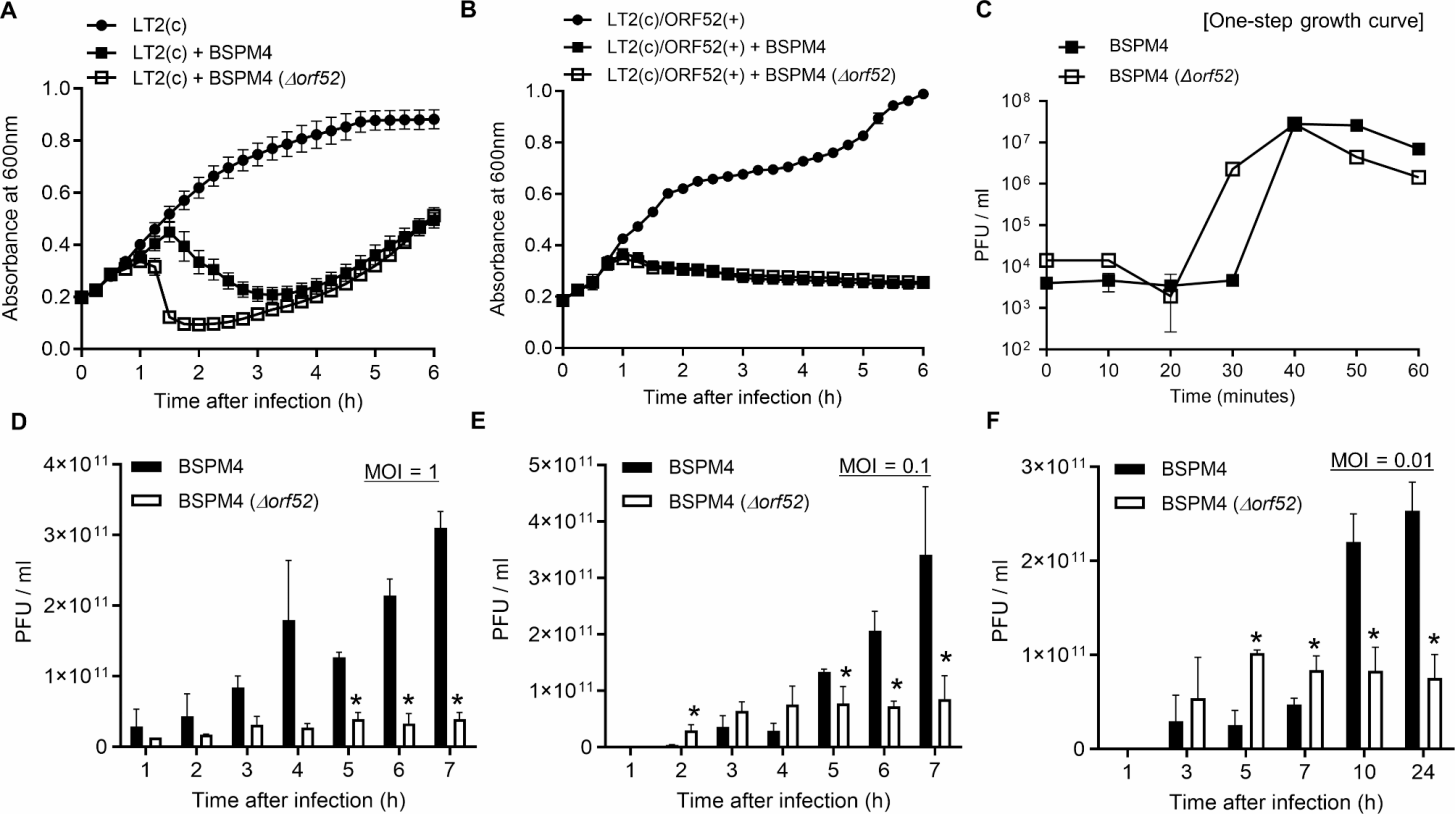
Role of *orf52* gene in phage infectivity and life cycle. (**A**) The cultures of *Salmonella* LT2(c) were infected with the wild-type or the mutant phage at an MOI of 0.1. (**B**) Bacterial cells harboring the pBAD-*orf52* were treated with arabinose and infected with phages at an MOI of 0.1. LT2(c)/ORF52(+) represents *Salmonella* LT2(c) expressing *orf52* (**C**) The replication cycles of mutant phage lacking *orf52* gene were compared with that of wild-type phage BSPM4. The *Salmonella* cells were infected with wild-type or mutant phage at MOI 1 (D), MOI 0.1 (**E**), and MOI 0.01 (**F**). A total number of produced phages was measured over time. An asterisk (*) denotes significance (*p* < 0.05, t-test) in the number of phages between the wild-type and mutant phages at the same time point

To investigate the physiological role of ORF52 in the phage life cycle, we constructed a mutant phage lacking the *orf52* gene (BSPM4 *Δorf52*) [25] (Fig. S5). The mutant phage caused bacterial cell lysis faster than the wild-type phage (Fig. 4A and Fig. S6). In addition, the plaques formed by the mutant phage were clearer and larger than those formed by the wild-type phage BSPM4 (Fig. S6D). However, the different lysis profile between wild-type and mutant phages disappeared when the cloned *orf52* gene was expressed in the host bacteria. (Fig. 4B). Both wild-type and mutant phages showed an efficiency of plating (EOP) of 10^− 2^ on host cells expressing the *orf52* gene (Fig. S6E).

Furthermore, one-step growth curve analysis revealed that the mutant phage lacking the *orf52* gene had a shorter latent period (20–30 min) than the wild type phage (30–40 min) even though burst sizes were not significantly different between the wild-type (52 ± 16 PFUs / cell) and the mutant phages (40 ± 15 PFUs / cell) (Fig. 4C). These results suggest that ORF52 could have an impact on controlling the timing of host lysis. Typically, holin controls the timing of phage-mediated lysis to optimize viral progeny production [26]. As shown in Fig. 3B, the expression of *orf52* inhibited the antibacterial activity of holin (ORF37). Therefore, the *orf52* deletion could result in faster bacterial cell lysis and shorter latent period of the mutant phage compared to the wild type phage probably due to full function of holin in the absence of the ORF52.

### Effects of the *orf52* on phage propagation

To explore the potential benefits of the *orf52* gene for phage propagation, we determined the total number of phages replicated from the wild-type and the mutant phage lacking the *orf52* gene. *Salmonella* cells were infected with each phage at an indicated multiplicity of infection (MOI), and the total number of reproduced phages was measured over 7 h. At an MOI 1.0, wild-type BSPM4 produced more infectious progeny compared to the mutant (Fig. 4D). At an MOI 0.1, the number of mutant phage particles was more than that of wild type during the first 4 h of phage replication. However, 5 h after infection, the number of phage particles from the wild-type phage was higher than that of the mutant (Fig. 4E). Similarly, at an MOI 0.01, the wild-type phage yielded more viral progeny than the mutant phage 10 h after infection (Fig. 4F). The one-step growth analysis revealed a shorter latent period of the mutant phage compared to the wild-type phage, implying that the mutant phage may produce more progeny. Nevertheless, these data indicate that the wild-type phage might have an advantage for phage propagation in the presence of high phage population.

### Lysis inhibition induced by superinfection of phage BSPM4

**Fig. 5:**
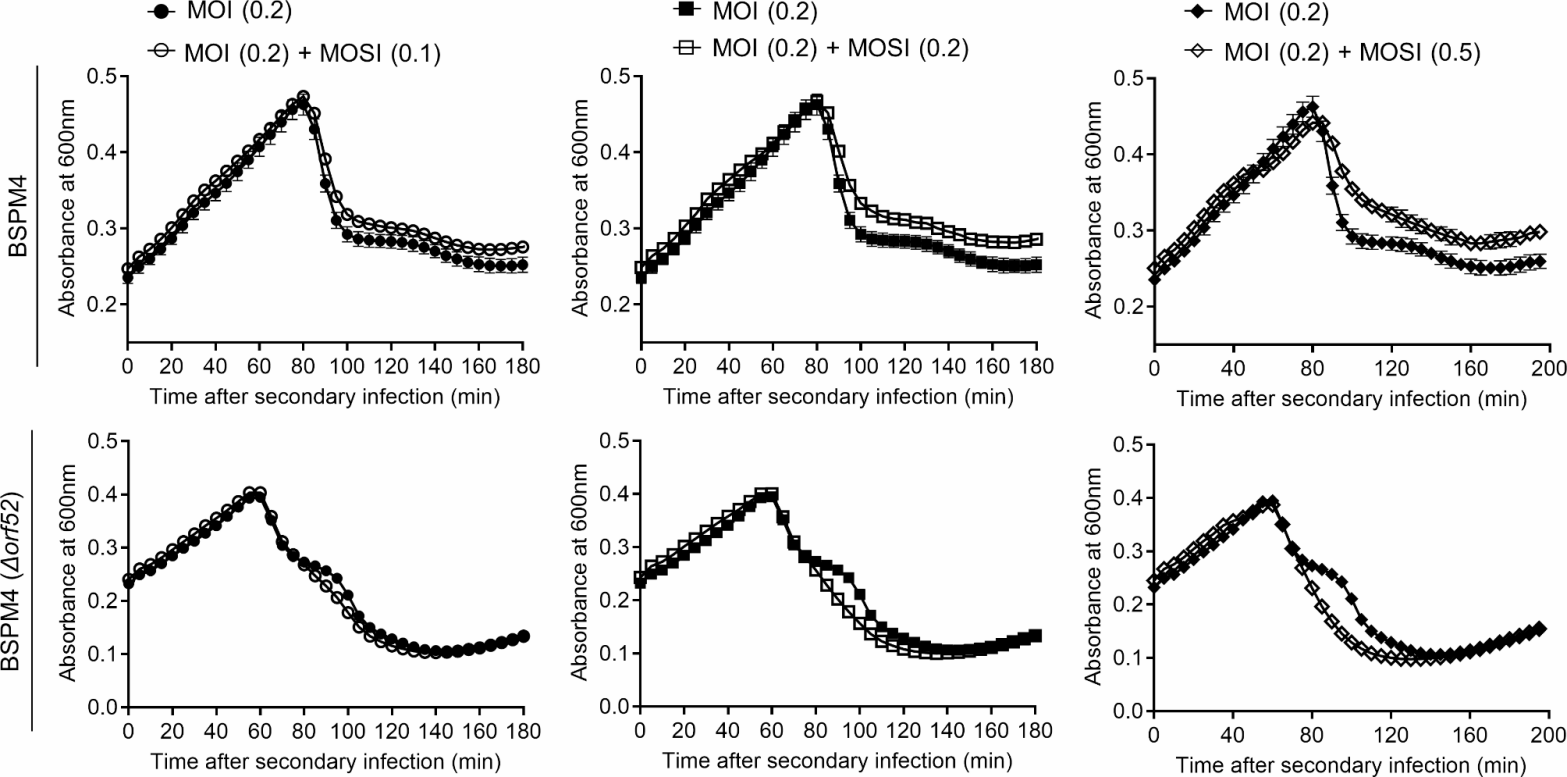
Lysis inhibition triggered by the phage protein ORF52. The effect of superinfection on phage lysis profile was determined by monitoring the absorbance at 600 nm. We infected *Salmonella* LT2(c) cells with wild-type or mutant phage at MOI 0.2 (multiplicity of infection) and incubate the culture at 37 ℃ for 15 min. Subsequently, the cultures were superinfected with the phages at the indicated MOSI (Multiplicity of superinfection), and the bacterial growth was monitored over time

Some phages (T-even phages) delay host cell lysis in response to superinfection, and this phenomenon is called lysis inhibition (LIN) [27, 28]. As the release of newly synthesized progeny is not advantageous when superinfection occurs, the phage replication cycle, particularly the latent period, is prolonged in the T4 phage [29]. LIN is one of phage systems that fine-tune infection parameters in response to host availability in the environment [30]. ORF52 does not exhibit amino acid sequence homology to proteins with known functions, but our results described above suggest that its function is similar to phage proteins (anti-holin) involved in LIN, specifically inhibiting the holin function, such as RI (from phage T4) [31, 32] and ArrA (from phage ICP1) [33]. Thus, we investigated whether LIN could be triggered by the superinfection in phage BSPM4. The *Salmonella* cells were infected with the phage. After 15 min, the cultures were divided into two groups. One group was subjected to superinfection with the phage, while the other group was treated with dilution buffer (Dulbecco’s phosphate-buffered saline). As shown in Fig. 5, superinfection with wild-type BSPM4 suppressed phage-mediated host cell lysis but superinfection accelerated host cell lysis in the case of the mutant phage.

### Timing of the *orf52* gene expression during the phage replication

**Fig. 6:**
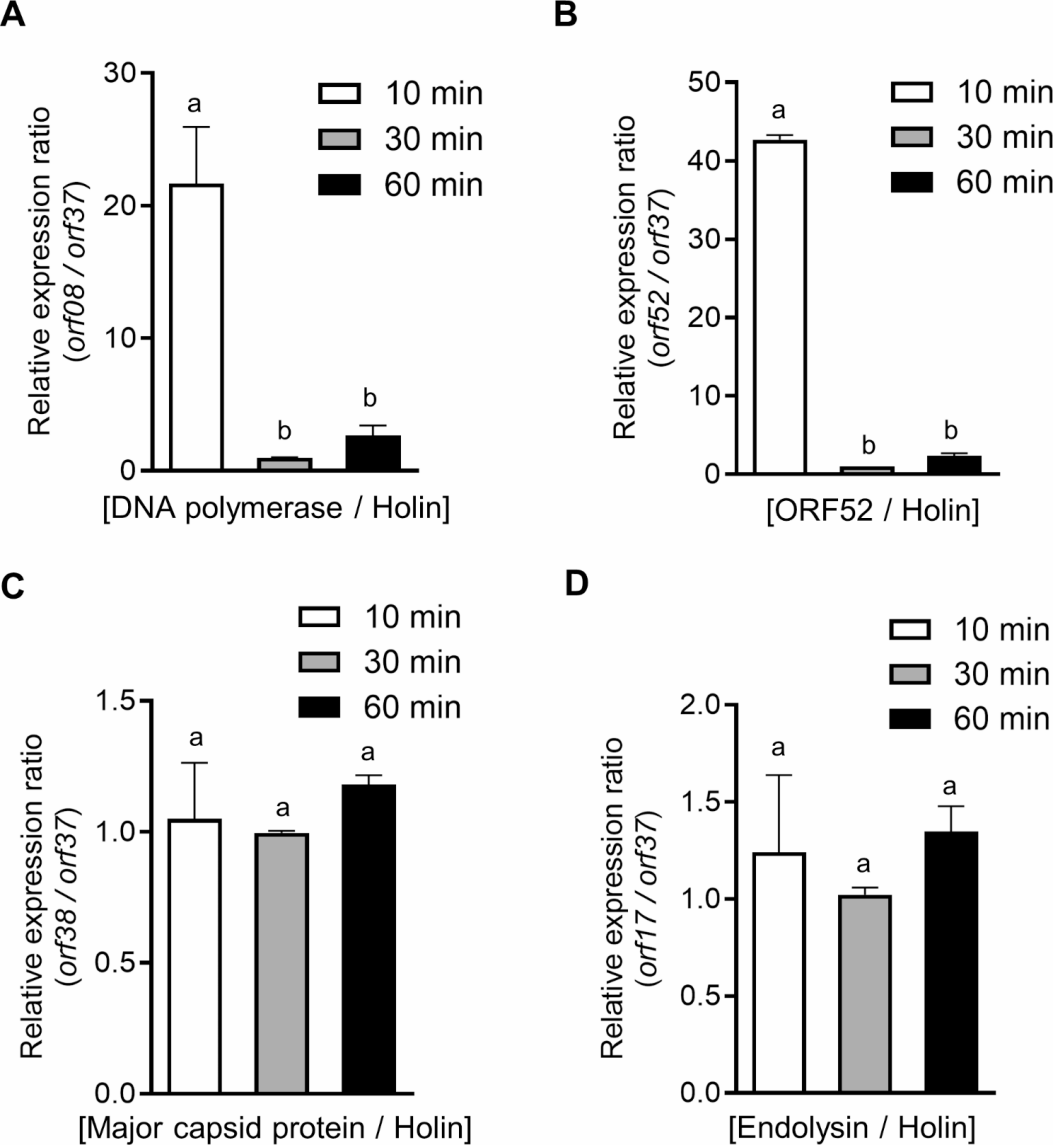
Gene expression pattern throughout phage life cycle. *Salmonella* cells were infected with phage BSPM4 and then after 10 min, 30 min, and 60 min of phage infection, a total RNA was isolated from phage-infected cells. Quantitative reverse transcription-polymerase chain reaction (qRT-PCR) assay was performed. The relative expression ratio of a target gene and to holin is calculated by following formula: *(relative expression level of a target gene* in a sample versus a control *in compare to reference gene) / (relative expression level of a holin in* sample versus a control *in compare to reference gene).* The expression of DNA polymerase (*orf08*) (**A**), *orf52 *(**B**), major capsid protein (*orf38*) (**C**), and endolysin (*orf17*) (**D**) was normalized to holin (*orf37*). 16S rRNA was used as a reference gene. Statistical comparisons between the groups were conducted using one-way ANOVA with the Bonferroni post-hoc test. The results of pairwise comparisons are summarized using the compact letter display (*p* < 0.05). Different letters indicate statistically significant differences (*p* < 0.05), while the same letters indicate no statistical difference

Viral gene expression is temporally regulated to optimize viral production and is divided into early and late phases [34–36]. The timing of gene expression during phage replication is crucial for gene function, providing insights into the mechanism of gene regulation. Based on the phage replication cycle (one-step growth analysis), total RNA was isolated from phage-infected cells at three-time points (10, 30, and 60 min) that span the entire replication cycle. The *orf08* (putative DNA polymerase) and *orf17* (putative major capsid protein) were respectively considered as ‘early gene’ [37]and ‘late gene’ [38], and these two genes were used as control genes to analyze the relative expression ratio of the *orf52* and the *orf37* (Fig. 6). The relative expression ratio patterns are categorized into two groups: the decreased group (*orf52* and *orf08*) and the consistent group (*orf38*, *orf40*, and *orf17*). The expression ratio of *orf08* (early gene) to holin was higher at 10 min than at 60 min while the ratio of *orf17* (late gene) to holin was not significantly changed, suggesting that holin can be categorized as a late gene. The pattern of the expression ratio of *orf52* to holin was similar to that of *orf08* (DNA polymerase, early gene). Moreover, at 10 min after infection, the raw Cp values of *orf52* were lower than those of holin in each sample, but at 60 min after infection, the raw Cp values of *orf52* were higher than those of holin (Fig. S7). These results propose that the *orf52* gene would be expressed earlier than holin in the phage replication cycle.

### Proposed mechanism of lysis inhibition in the phage BSPM4

**Fig. 7:**
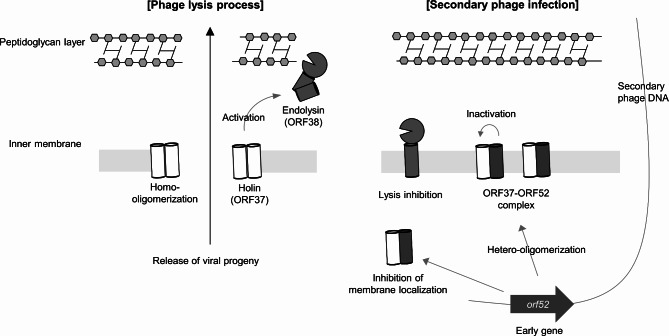
Proposed model of lysis inhibition and the role of ORF52 in the phage replication cycle. In a superinfection, the DNA of a secondary infecting phage is injected into the bacterial cytoplasm, leading to the rapid synthesis of the phage protein ORF52 (early gene product). This ORF52, synthesized from the secondary phage, localizes to the bacterial membrane and binds to the holin (ORF37) produced by the primary phage. This interaction between ORF52 and holin can inhibit phage-mediated cell lysis

Based on the above results, we propose a molecular mechanism for the ORF52-mediated lysis inhibition (LIN) triggered by the superinfection of phage BSPM4 (Fig. 7). Protein ORF52 may serve as a signal for secondary infection by surrounding phages and simultaneously regulate phage-mediated cell lysis by suppressing the function of holin. In primary infection, phages replicate within host bacteria and produce their lysis cassette proteins to release viral progeny. However, if superinfection occurs during phage replication, intracellular concentration of ORF52 will be increased because the early gene, *orf52*, is expressed additionally from the superinfecting phage. Higher intracellular concentration of ORF52 can inhibit the function of holin produced by the primary infecting phage, leading to a delay in cell lysis. Superinfections indicate a reduced number of host bacteria available in the environment for viral progeny. We observed that ORF52 could confer replication advantages to the phages under high phage density conditions (Fig. 4D-F). In environments where the ratio of phages to bacteria is high, phages encoding the *orf52* gene could have a replication advantage by delaying host cell lysis to ensure a sufficient bacteria population for progeny phage replication. Superinfection occurs when the number of phages exceeds the number of bacteria. Therefore, the phage-to-bacteria ratio is a crucial factor in determining the replication advantages conferred by ORF52. BSPM4-like phages that possess a gene homologous to *orf52* exhibit protein sequence homology with the lysis cassette of BSPM4 (Fig. S8). This homology suggests that BSPM4-like phages may employ a lysis inhibition (LIN) mechanism as an effective strategy to optimize phage production by coordinating the timing of cell lysis.

### Biological importance of lysis inhibition

In phage-host interactions, phages employ various evolutionary strategies to sense bacterial populations in the environment. For example, *Bacillus* phage SPβ utilizes a peptide-based communication system called arbitrium to coordinate the lysis-lysogen decision [39]. *Vibrio* phage VP882 can utilize host-produced quorum-sensing autoinducer molecules to regulate its lysis-lysogeny decision [40]. LIN is another type of phage system that adjusts lysis time based on the number of bacteria in its environment. The holin-anti-holin mechanism represents a sophisticated strategy employed by bacteriophages to regulate the lytic cycle and maximize their reproductive success within bacterial hosts. Understanding the dynamics of this mechanism is important not only for deciphering the molecular details of phage-host interactions but also for potential applications in the phage therapy. Our discovery of ORF52 in the *Salmonella* phage provides a unique example of a phage protein involved in LIN and highlights the significance of phages fine-tuning their infection parameters in response to the availability of hosts in the environment. However, the detailed molecular mechanism underlying this antibacterial process is not fully elucidated. Further research is required to understand how ORF52 interacts with membrane components and its impact on cellular processes.

## Conclusion

Phages, the most abundant biological entities on Earth, play a crucial role in bacterial ecology and evolution through their host specificity and lytic activities. Given their vast numbers and significant ecological impact, understanding the functions of phage genes is essential for their effective use as biocontrol agents. In this study, we explored the genome of phage BSPM4 and identified a gene, *orf52*, with the potential to modulate holin function. ORF52 acts as a regulatory element, optimizing viral production by coordinating the timing of cell lysis and providing replication advantages to phages. Our discovery of ORF52’s role in the *Salmonella* phage reveals the sophisticated replication strategies employed by phages.

## Electronic supplementary material

Below is the link to the electronic supplementary material.

Supplementary Material 1

## Data Availability

The complete genome sequence of phage BSMP4 was deposited in NCBI’s Genbank and is available via accession number NC_048655. The accession number for protein ORF52 is AQY55228.

## Abbreviations

SAR: Signal-Arrest-Release
SEC: Secretory
ORF: Open Reading Frame
WT: Wild Type
TEM: Transmission Electron Microscopy
EOP: Efficiency Of Plating
MOI: Multiplicity Of Infection
MOSI: Multiplicity Of Superinfection
LIN: Lysis Inhibition
LB: Luria-Bertani
Km: Kanamycin
Cm: Chloramphenicol
IPTG: Isopropyl-β-thiogalactopyranoside
Ap: Ampicillin
EDTA: Ethylenediamine-Tetraacetic Acid
SDS-PAGE: Sodium Dodecyl Sulfate-Polyacrylamide Gel Electrophoresis
Ni-NTA: Ni-Nitrilotriacetic Acid
PFU: Plaque Forming Unit
qRT-PCR: quantitative Reverse Transcription-Polymerase Chain Reaction
