# Supplementary material for: Elucidation of molecular function of phage protein responsible for optimization of host cell lysis
Source: BMC Microbiol. 2024 Dec 19;24:532. doi: 10.1186/s12866-024-03684-9 (PMC11657625; doi:10.1186/s12866-024-03684-9)
Supplement: Supplementary file 1 — Supplementary Material 1 [file 12866_2024_3684_MOESM1_ESM.pdf]

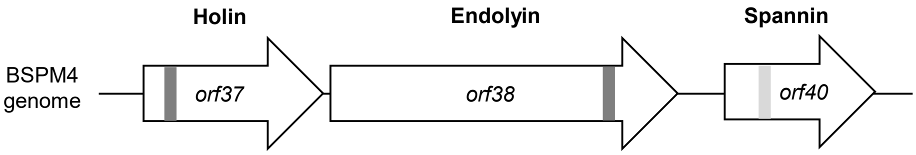

Fig. S1. Schematic representation of the phage BSPM4 lysis cassette. The phage BSPM4 genome was predicted to encode three lysis genes, including holin (*orf37*), endolysin (*orf38*), and spanin (*orf40*). The transmembrane domain was predicted by the TMHMM 2.0 softerware. Lipoprotein signal peptide were respectively predicted by LipoP 1.0 Server. The sites of potential transmembrane domain are indicated by grey bar (in ORF37 and ORF38), and lipoprotein signal peptide is indicated by light grey bar (in ORF40).

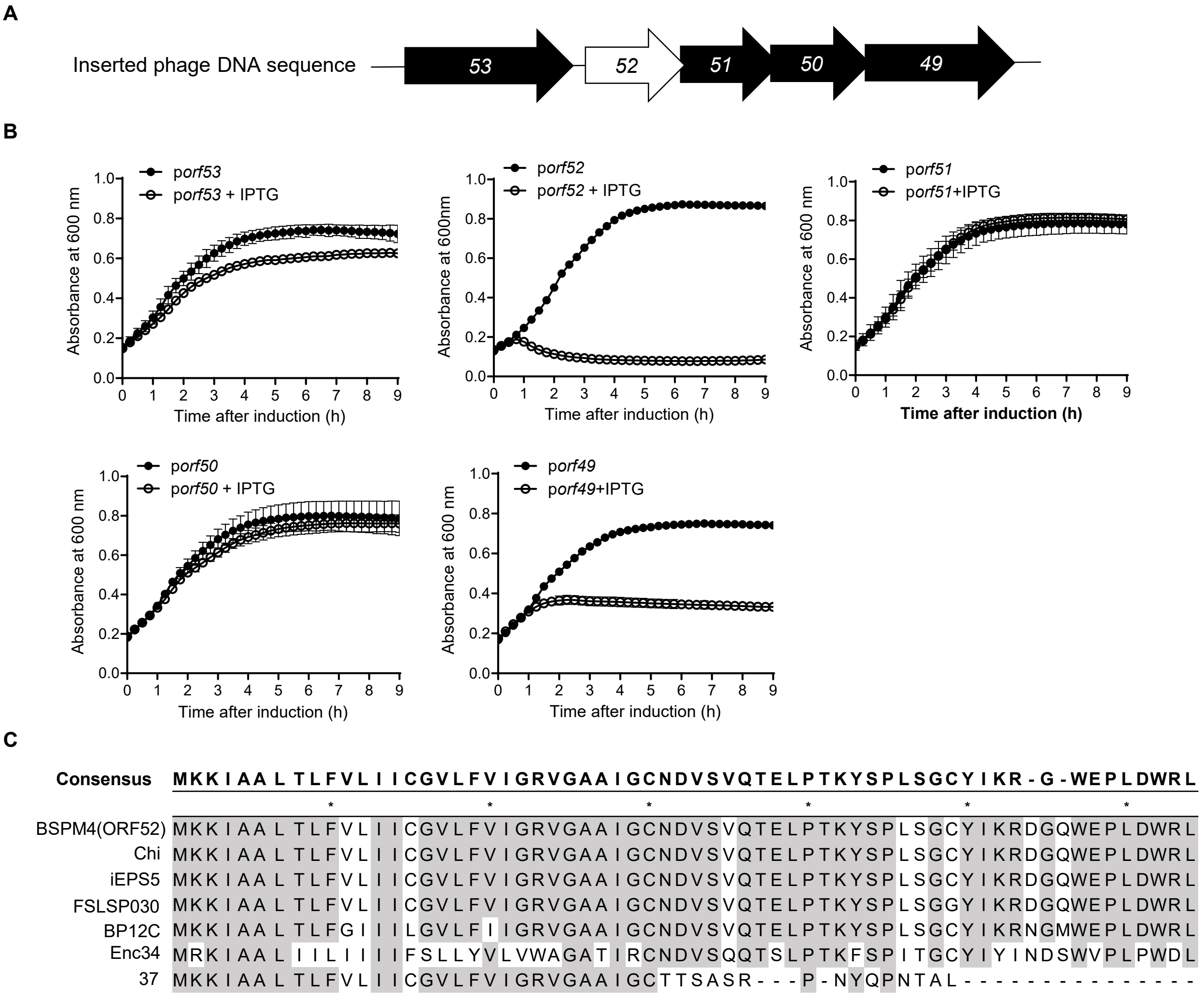

Fig. S2. Identification of antibacterial protein ORF52 in phage BSPM4 genome. One clone showing strong growth inhibition upon gene induction was selected from a random phage DNA library. (A) The plasmid from the clone was purified and inserted phage DNA was sequenced. The plasmid encodes five open reading frames (ORFs) including *orf53*, *orf52*, *orf51*, *orf50*, and *orf49*. (B) To identify the gene(s) responsible for bacterial inhibition, each gene was cloned into an expression vector (pUHE21-2-*lacI*^{q}). The effect of each gene expression on bacterial growth was determined. (C) Multiple sequence alignment of ORF52 family from various phage sources was performed. The amino acid sequences of phage BSMP4, Chi, iEPS5, FSLSP030, BP12C, Enc34, and 37 were used. Every tenth residue is marked by an asterisk. Strictly (80%) conserved residues are highlighted in grey.

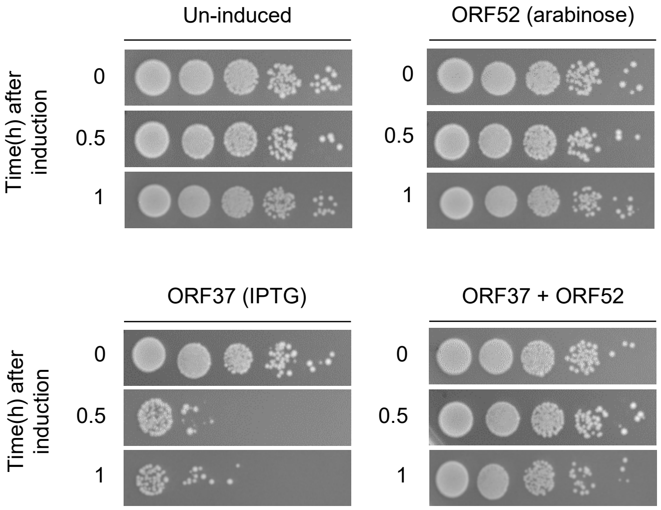

Fig. S3. Interaction between phage protein ORF52 and ORF37. The number of viable *Salmonella* harboring plasmid with ORF37 (pUHE21::*orf37*) and ORF52 (pBAD33::*orf52*) was determined by counting colonies on LB agar plate. The expression of *orf52* was induced by 0.02% arabinose, and *orf37* was induced by 0.5 mM IPTG. Bacterial cells expressing *orf52* or *orf37* were designated as ORF52 or ORF37. The cell expressing both *orf52* and *orf37* was designated as ORF52 + ORF37.

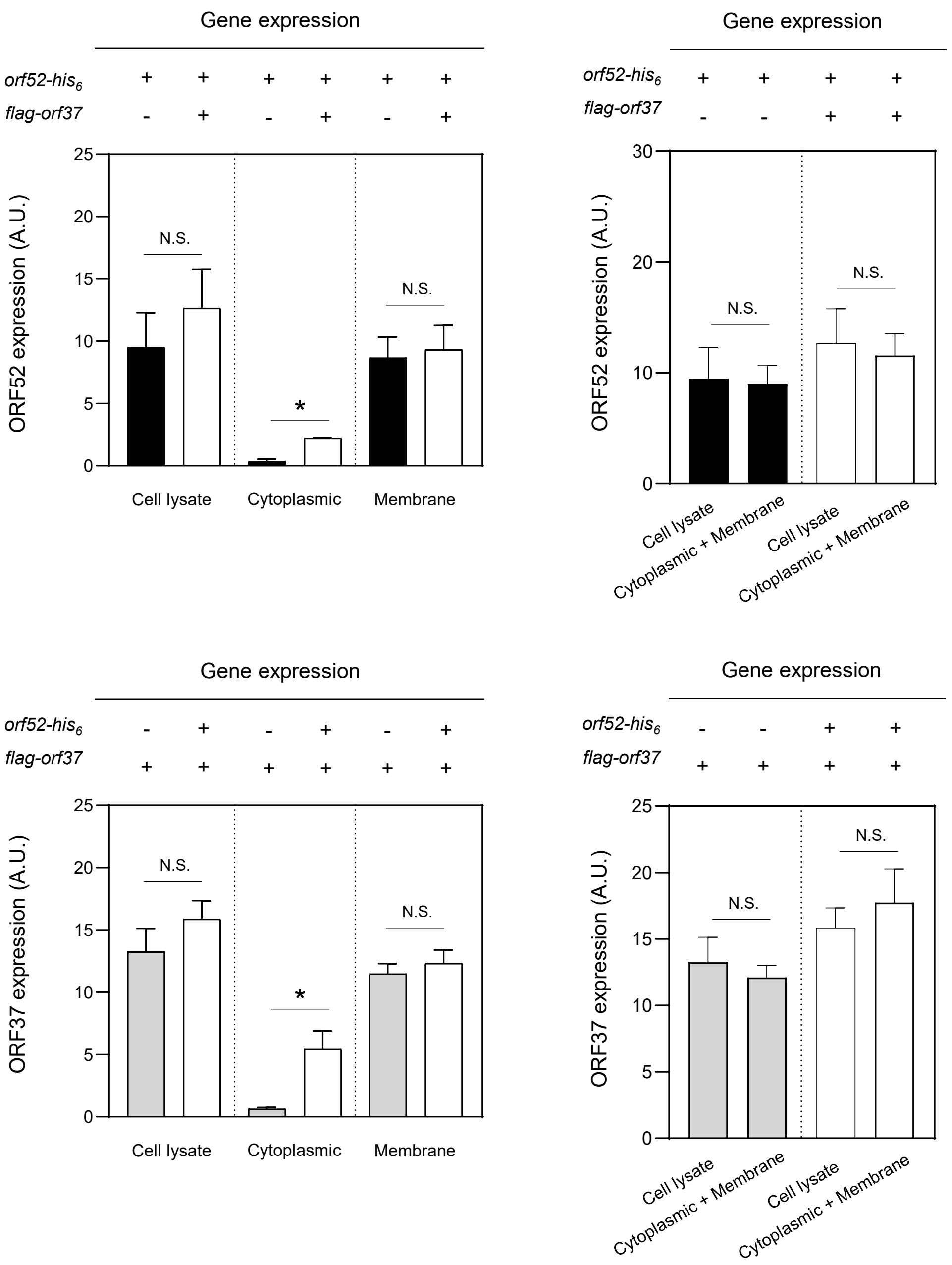

Fig. S4. Effect of ORF52 on the localization of holin. *Salmonella* Typhimurium cells harboring two plasmids encoding an N-terminal FLAG-tagged ORF37 (pUHE1::*flag-orf37*) and a C-terminal His-tagged ORF52 (pBAD33::o*rf52-his*$_6$) were cultured. After ORF52 expression for 20 minutes, ORF37 was subsequently induced for 20 minutes. The cultures were fractionated into cytoplasmic and membrane fractions. Target protein expression was measured by Western blot, and the relative band intensity was quantified using the Image J software (n=3). An asterisk (*) denotes a significant difference at $p < 0.05$, while 'N.S.' indicates non-significance.

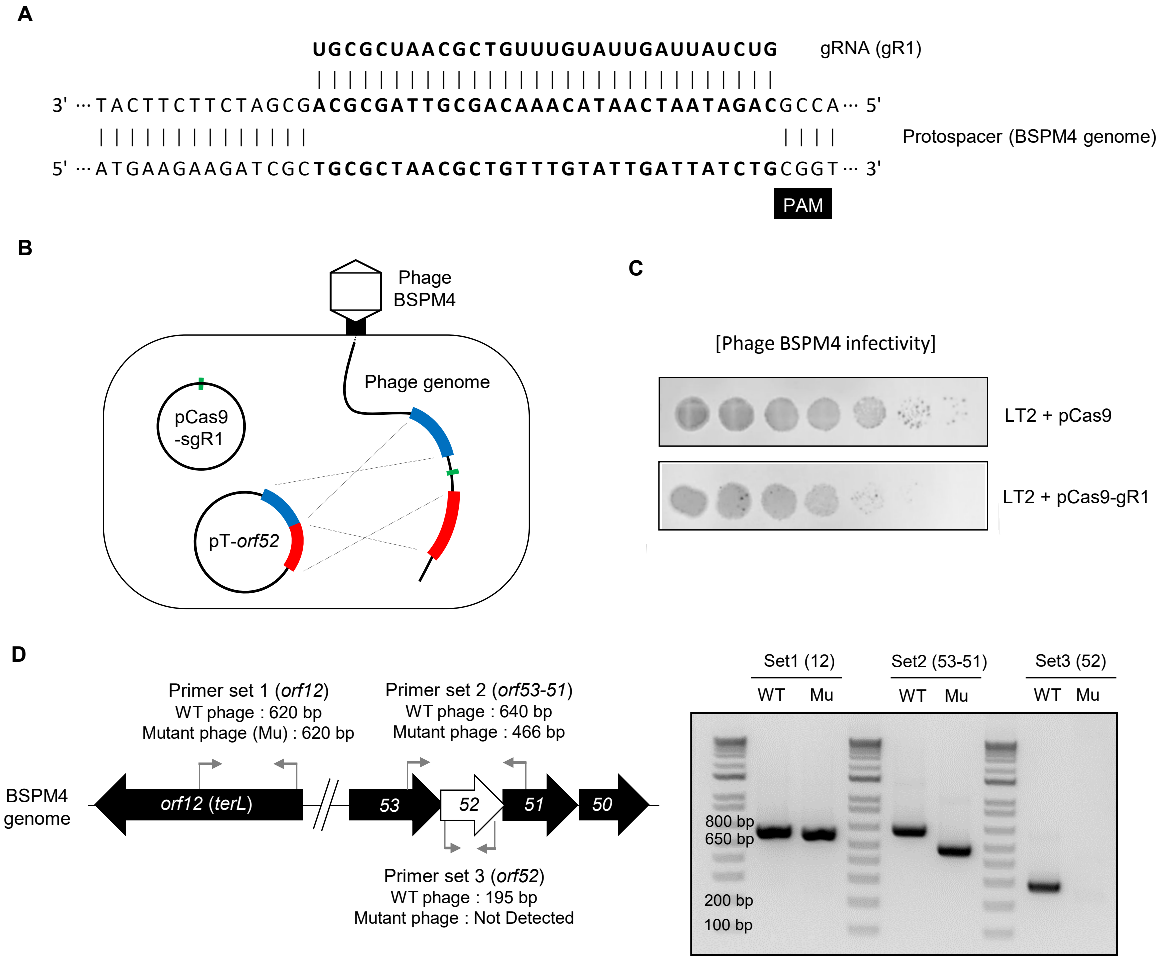

Fig. S5. Construction of mutant phage lacking *orf52* using CRISPR-Cas9 system. (A) Guide RNA sequence was designed by considering the position of protospacer adjacent motif (PAM) sites in the target region of *orf52* gene. (B) Schematic strategy of the CRIPSR-Cas9 associated phage genome editing strategy is shown. The host bacteria carrying plasmids (pCas9-sgR1 and pT-*orf52*) were infected with phage BSPM4. The plasmids pCas9-sgR1 was used for cleaving a target DNA (wild-type BSPM4) and the plasmid pT-*orf52* was used for donor DNA that contains homology arms (500 bp) flanking the cleavage site. The green bar indicates the target cleavage sites and the homology arms are represented as red and blue bar (C) Efficiency of plating (EOP) was determined to investigate whether the CRIPSR-Cas9 system work on the phage BSPM4. The 10-fold serially diluted phages were spotted on lawns of wild-type *Salmonella* and *Salmonella* harboring CRISPR-Cas9 components. (D) The PCRs were performed to confirm the deletion of *orf52* gene in phage BSPM4.

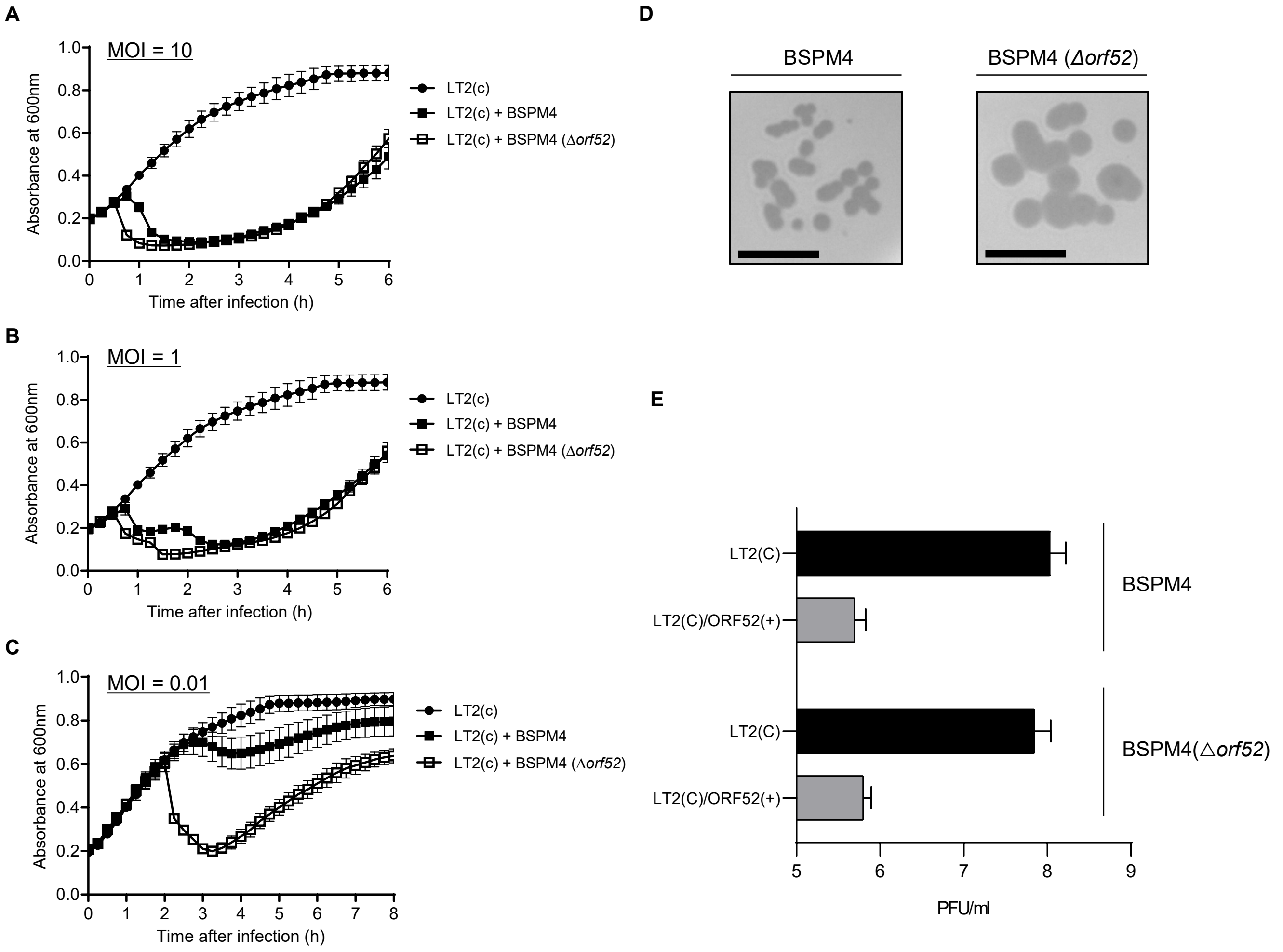

Fig. S6. Characterization of phage BSPM4 and mutant phage lacking *orf52* gene. The culture of exponentially growing *S.* Typhimurium LT2(c) were treated with phage(s) at an indicated multiplicity of infection (MOI), and the mixtures were aerobically incubated at 37°C. The absorbance at 600nm was monitored every 15 min (A-C). The culture of overnight host bacteria was added to soft LB agar (0.3% agar, final concentration), and the mixture was poured on a LB agar plate. Serially diluted phages were spotted onto the soft LB agar layer and incubated at 30°C for 12 hours. The data are the representative results of three independent experiments showing similar plaque morphology. Scale bars (black line in lower left corner) represent 1 cm (D). Efficiency of plating (EOP) was determined by comparing the number of plaques formed by the phage BSPM4 on the wild-type host [LT2(c)] and bacteria expressing *orf52* [LT2(c)/ORF52(+)] (E).

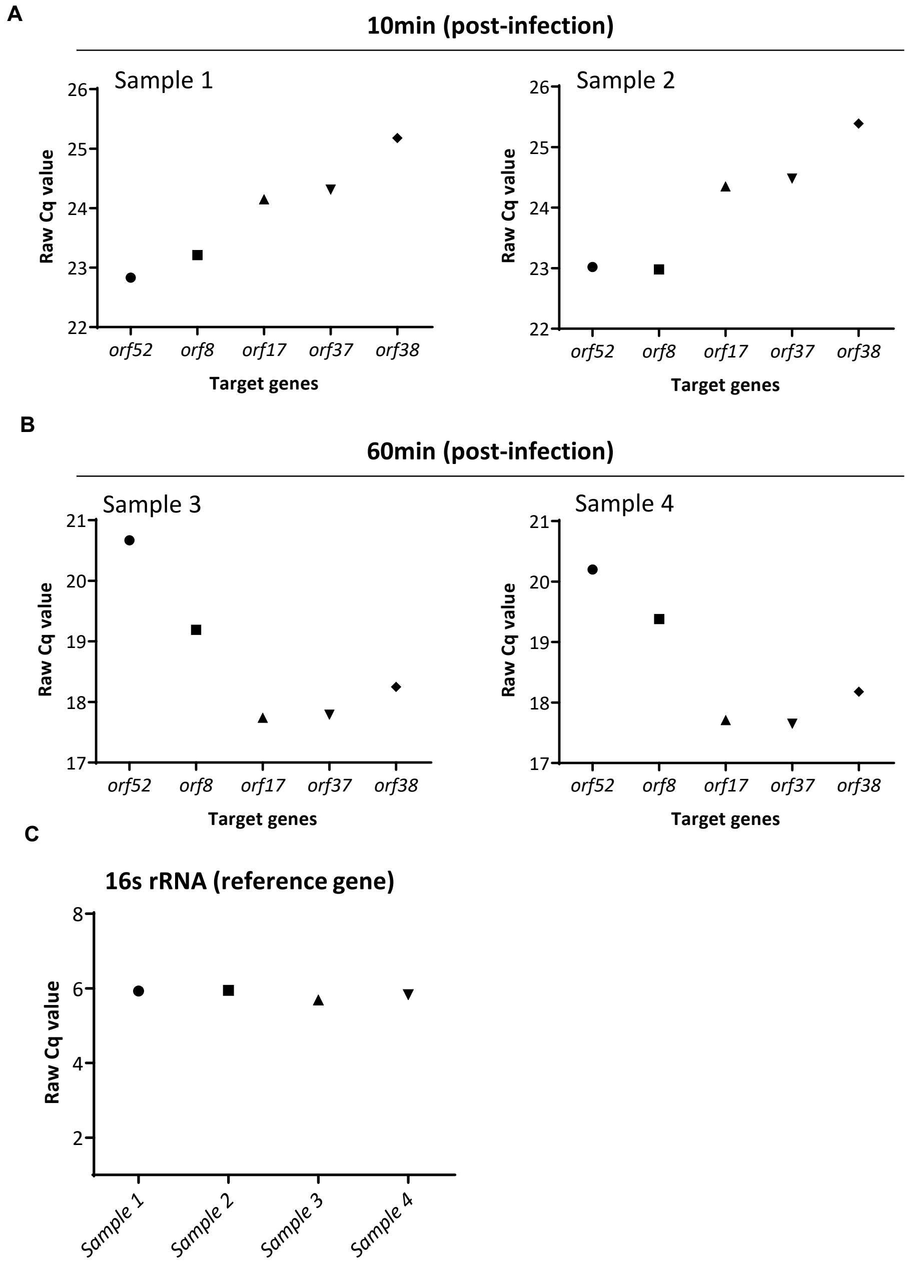

Fig. S7. Raw Cq values. *Salmonella* cells were infected with phage BSPM4 and then after 10 min (A), and 60 min (B) of phage infection, a total RNA was isolated from phage-infected cells. cDNA was synthesized from 2 µg total RNA and quantitative reverse transcription-polymerase chain reaction (qRT-PCR) assay was performed. For each sample, the raw Cq values of *orf52*, *orf8*, *orf17*, *orf37*, and *orf38* were compared. 16s rRNA gene was used as an internal control (C).

A Holin

```
                             20                  40                  60                  80                  100
BSPM4    : MSEMERERPGDNSGGIKLDLSVNLPTILTMASMLVAAVLYVNNRFADLSNQNTQTDTRLTNVEKRQDSTDAAFTVLRAETVSQNSALRSDLRADMRDLKQSVDTLSSQMMRK-- : 112
Chi      : MSEMERERPGDNSGGIKLDLSVNLPTILTMASMLVAAVLYVNNRFADLSNQNTQTDTRLTNVEKRQDSTDAAFTVLRAETVSQNSALRSDLRADMRDLKQSVDALSSQMMRK-- : 112
iEPS5    : MSEMERERPGDTSGGIKLDLSVNLPTILTMASMLVAAVLYVNNRFADLSNQNTQTDTRLTNVEKRQDSTEAAFTVLRAETVSQNSALRSDLRADMRDLKQSVDTLSSQMMRK-- : 112
FSLSP030 : MSEMERERPGDNSGGIKLDLSVNLPTILTMASMLVAAVLYVNNRFADLSNQNTQTDTRLTNVEKRQDSTDAAFTVLRAETVSQNSALRSDLRADMRDLKQSVDTLSSQMMRK-- : 112
BP12C    : MSDMERERPDDNSGGIKLDLSVNLPTILTMASMLVAAVLYVNNRFADLSNQNTQAETRLTNVEKRQDATDAAFTVLRAETVSQNSALRSDLRADMRDLKQSVDTLSSQMMRK-- : 112
Enc34    : MSDMERDRENEGTGGIRLDLSLNIPTILLMTTMLVSSVLYLNNRFGELQSQSVQVDTRLTNVEKRQDNFDTAITIVRTEASASNAALRQDVRADIRDVKNSVDTLTATLNSRGR : 114
37       : MSEMERERPGDNSGGIKLDLSVNLPTILTMASMLVAAVLYVNNRFADLSNQNTQTDTRLTNVEKRQDSTDAAFTVLRAETVSQNSALRSDLRADMRDLKQSVDTLSSQMMRK-- : 112
           MS MERERP D SGGIKLDLSVNLPTILTMASMLVAAVLYVNNRFADLSNQNTQ DTRLTNVEKRQD TDAAFTVLRAETVSQNSALRSDLRADMRDLKQSVDTLSSQMMRK
```

B Endolysin

```
                             20                  40                  60                  80                 100                 120
BSPM4    : MAKQ-KPRGIRNNNPGNIEWGSPWQGLIPRNEATDSRFAQFKDPASGIRAIAVTLTTYYDKRKANDGSKIDSVREVIERWAP-----AVENNVSAYAKQVAAVLAVDPNSEMLNLHDYDTMRGLVEGIIR : 124
Chi      : MAKQ-KPRGIRNNNPGNIEWGSPWQGLIPRSEATDSRFAQFKDPASGIRAIAVTLTTYYDKRKANDGSKIDSVREVIERWAP-----AVENNVSAYAKQVAAVLDVNPNSETLNLHDYDTMRGLVEGIIR : 124
iEPS5    : MAKQ-KPRGIRNNNPGNIEWGSPWQGLIPRNEATDSRFAQFKDPASGIRAIAVTLTTYYDKRKANDGSKIDSVREVIERWAP-----AVENNVSAYAKQVAAVLAVDPNSETLNLHDYDTMRGLVEGIIR : 124
FSLSP030 : MAKQ-EPRGIRNNNPGNIEWGSPWQGLIPRSEATDSRFAQFKDPASGIRAIAVTLTTYYDKRKANDGSKIDSVREVIERWAP-----AVENNVSAYAKQVAAVLDVNPNSETLNLHDYDTMRGLVEGIIR : 124
BP12C    : MAKQ-KPRGIRNNNPGNIEWGSPWQGLIPRNQATDNRFAQFKDPASGIRAIAVTLTTYYDKRKANDGSKIDSVREVIERWAP-----AVENNVSAYAKQVAAVLAVDPNSETLNLHDHDTMRGLVEGIIR : 124
Enc34    : MAKTSLPRGIRNNNPGNIEWGSPWQGLQARTAASDPRFCQFIDPASGIRALAVILTTYFDKRKAADGSKIDTIREVIERWAPPKKNGVVENNTTAYANQIARVLNMQPDDETLNLHDYETMRKMVEGIIR : 130
37       : M---------------------------------------------------------------------------IERWAP-----AVENNVSAYAKQVAAVLAVDPNSETLNLHDHDTMRGLVEGIIR :  50
           MAK   PRGIRNNNPGNIEWGSPWQGL  R  A D RF QF DPASGIRA AV LTTY DKRKA DGSKID  REVIERWAP     AVENNVSAYAKQVAAVL V PNSETLNLHD DTMRGLVEGIIR

                        140                 160                 180                 200                 220                 240
BSPM4    : HENGNPEAFGLTPYSNANTWYSDEVIEEGLRRAGIVKAAKPVNRTTVAATSVAGLGAAQLVDMVQPVKAAMDSAHGDISSGDWVRIAFGAATIAIGLYMGWVAYRKHRAGAAA : 237
Chi      : HENGNPESFGLTPYNNENTWYSDEVIEEGLRRAGIVKAAKPVNRTTVAATSVAGLGAAQLVDMVQPVKAAMDSAHGDISSGDWVRIAFGAATIAIGLYMGWVAYRKHRAGAAA : 237
iEPS5    : HENGNPEAFGLTPYSNANMWYSDEVIEEGLRRAGIVKAAKPVNRTTVAATSVAGLGAAQLVDMVQPVKAAMDSAHGDISSGDWVRIAFGAATIAIGLYMGWVAYRKHRAGAAA : 237
FSLSP030 : HENGNPEAFGLTPYNNANTWYSDEVIEEGLRRAGIVKATKPVNRTTVAATSVAGLGAAQLVDMVQPVKAAMDSAHGDISSGDWVRIAFGAATIAIGLYMGWVAYRKHRAGAAA : 237
BP12C    : HENGNPASFGLTPYSNANTWYSDEVIEEGLRRAGIVKAAKPVNRTTVAATSVAGLGAAQLVDMVQPVKAAMDSAHGDISSGDWVRIAFGAATIAVGLYMGWVAYRKHRAGAAA : 237
Enc34    : HENGSPEDYDRAPYNNINQWYSDEQIAEGLRRAGLVKPKTAVNRATVTATSVTGLGAAQLVDLVQPVKAAMDSAHGDISSGDWVRIAFGVMTIGVGLYMGWVAYQKHKAGAA- : 242
37       : HENGNPASFGLTPYSNANTWYSDEVIEEGLRRAGIVKAAKPVNRTTVAATSVAGLGAAQLVDMVQPVKAAMDSAHGDISSGDWVRIAFGAATIAVGLYMGWVAYRKHRAGAAA : 163
           HENGNP  FGLTPY N N WYSDEVIEEGLRRAGIVKA KPVNRTTVAATSVAGLGAAQLVDMVQPVKAAMDSAHGDISSGDWVRIAFGAATIA GLYMGWVAYRKHRAGAAA
```

Fig. S8. Multiple sequence alignments of lysis cassette proteins. Protein sequences of holin (A) and endolysin (B) from phages BSPM4, Chi, iEPS5, FSLSP030, BP12C, Enc34, and 37 were aligned using ClustalX2. Strictly (80%) conserved residues are highlighted in grey.

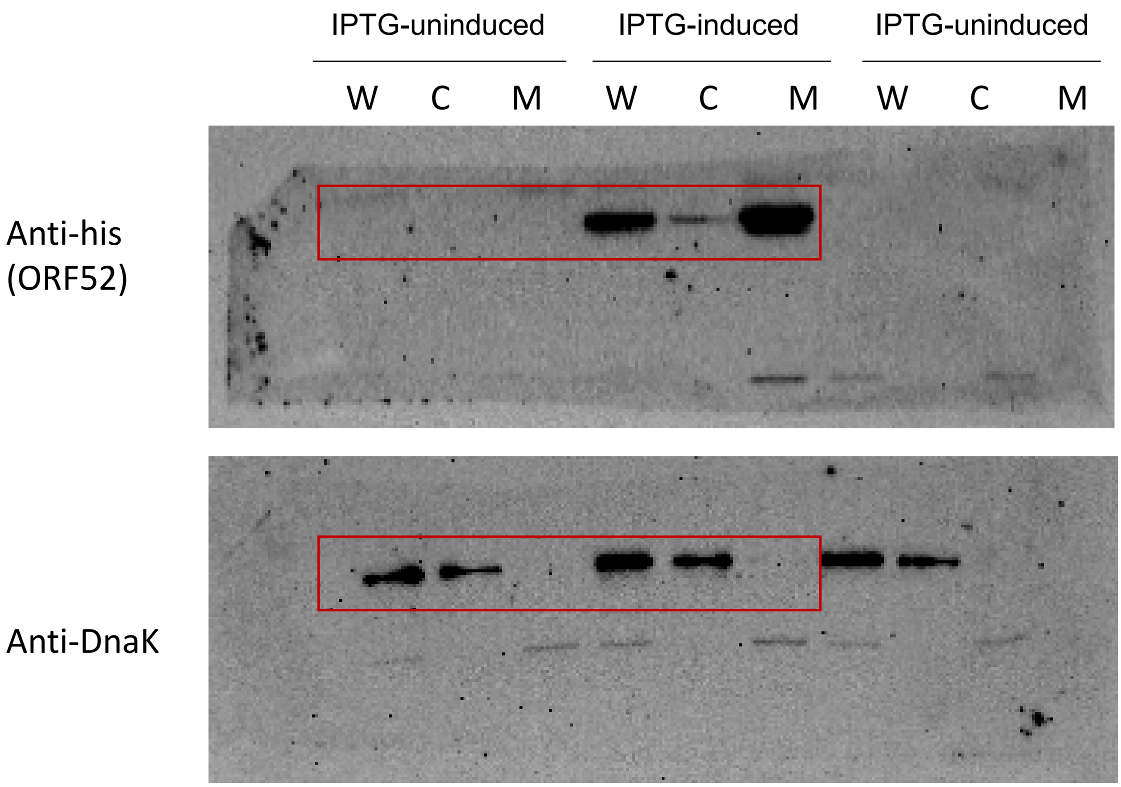

Fig. S9. Full-length western blot analysis of ORF52 localization in different cellular fractions. Bacterial cells harboring the plasmid encoding a C-terminal His-tagged ORF52 (pUHE21::*orf52-hisx6*) were cultured and fractionated into whole cell lysate [W], cytoplasmic [C], and membrane [M] fractions. The expression levels of ORF52 were analyzed by Western blot using an anti-His antibody. DnaK was used for cytoplasmic fractions. The cropped gel data are presented in Fig. 1D. The key sections in the main figure are marked with red rectangular boxes.

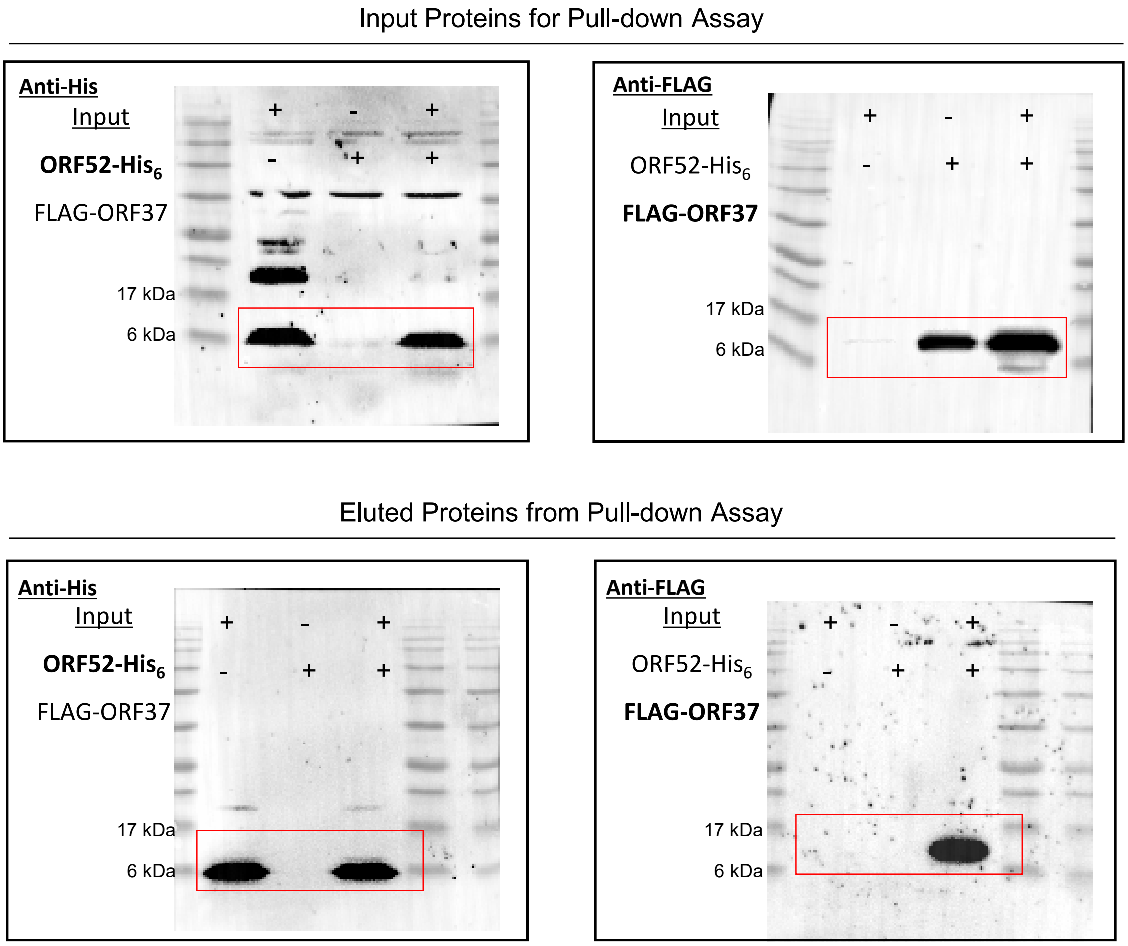

Fig. S10. Analysis of input and eluted proteins from pull-down assay. Input proteins (top panels) and eluted fractions (bottom panels) were analyzed using anti-His and anti-FLAG antibodies. The cropped gel data are presented in Fig. 3E. The key regions in the main figure are highlighted with red rectangular boxes.

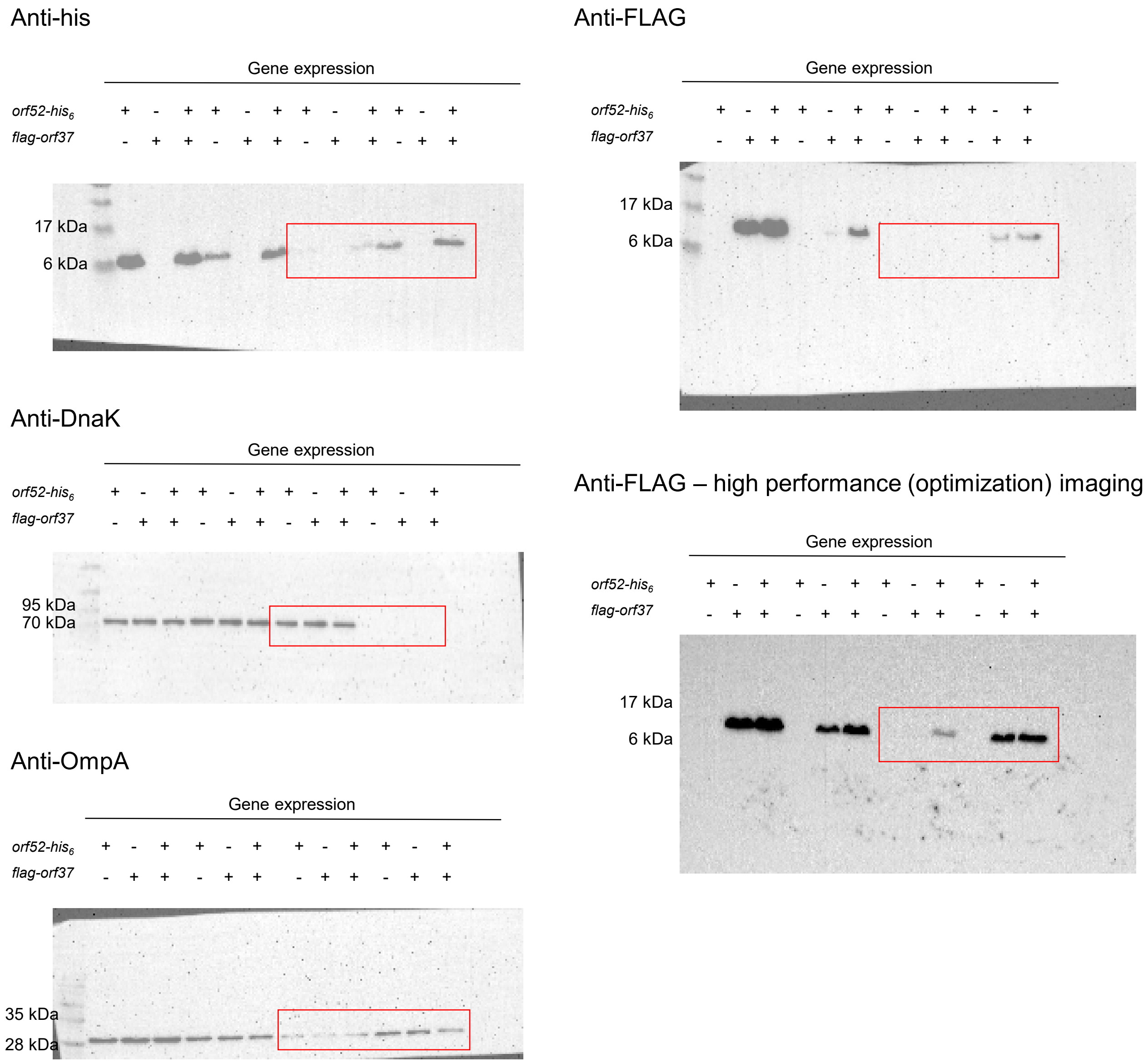

Fig. S11. Localization of ORF52 and ORF37 upon gene expression. Bacterial cells were fractionated into cytoplasmic and membrane fractions under different gene expression. Anti-DnaK and anti-OmpA antibodies were used as controls for the cytoplasmic and membrane fractions, respectively. The cropped gel data are presented in Fig. 3F. The key regions in the main figure are highlighted using red rectangular boxes.

Table S1. Plasmids used in this study

Plasmids	Characteristics	Source
**pUHE21-2-*lacI^q***	*pMB1 ori*, *lac* promoter (IPTG inducible expression plasmid), *lacIq Ap^R*	
pA1(Plate1)	pUHE21-2-*lacI::orf53,52,51,50, and 49*	In this study
p*orf53*	pUHE21-2-*lacI::orf53*	In this study
p*orf52*	pUHE21-2-*lacI^q::orf52*	In this study
p*orf51*	pUHE21-2-*lacI^q::orf51*	In this study
p*orf50*	pUHE21-2-*lacI^q::orf50*	In this study
P*orf49*	pUHE21-2-*lacI^q::orf49*	In this study
p*orf37*	pUHE21-2-*lacI^q::orf37*	In this study
p*orf38*	pUHE21-2-*lacI^q::orf38*	In this study
p*orf40*	pUHE21-2-*lacI^q::orf40*	In this study
p*orf52-his*$_{x6}$	pUHE21-2-*lacI^q::orf52-his*$_{x6}$(C-terminal hexahistidine-tagged ORF52)	In this study
p*flag-orf37*	pUHE21-2-*lacI^q::flag-orf49* (N-terminal flag-tagged ORF37)	In this study
**pBAD33**	*p15A ori,* L-arabinose-inducible expression plasmid*, araC, Cm^R*	
pBAD-*orf52*	pBAD33::*orf52*	In this study
pBAD-*orf52-his*$_{x6}$	pBAD33::*orf52-his*$_{x6}$(C-terminal hexahistidine-tagged ORF52)	In this study
**pCas9**	*p15A ori, Cas9 (from Streptococcus pneumoniae), tracrRNA, Cm^R*	
pCas9-sgR1	*pCas9 with single guide RNA1 (targeting orf37)*	In this study
**pT**	*pMB1 ori, Km^R*	
pT-*orf52*	*pT with 500 bp of homology to the regions upstream and downstream of orf52*	In this study

Ap^R, ampicillin resistant; Km^R, kanamycin resistant; Cm^R, chloramphenicol resistant
